# Genomic variation in two gametocyte non-producing *Plasmodium falciparum* clonal lines

**DOI:** 10.1186/s12936-016-1254-1

**Published:** 2016-04-21

**Authors:** Susana Campino, Ernest Diez Benavente, Samuel Assefa, Eloise Thompson, Laura G. Drought, Catherine J. Taylor, Zaria Gorvett, Celine K. Carret, Christian Flueck, Al C. Ivens, Dominic P. Kwiatkowski, Pietro Alano, David A. Baker, Taane G. Clark

**Affiliations:** Faculty of Infectious and Tropical Diseases, London School of Hygiene & Tropical Medicine, London, UK; The European Molecular Biology Organization, Heidelberg, Germany; Centre for Immunity, Infection and Evolution, University of Edinburgh, Edinburgh, UK; Wellcome Trust Centre for Human Genetics, University of Oxford, Oxford, UK; Wellcome Trust Sanger Institute, Hinxton, Cambridgeshire UK; Dipartimento di Malattie Infettive, Parassitarie ed Immunomediate, Istituto Superiore di Sanità, Rome, Italy; Faculty of Epidemiology and Population Health, London School of Hygiene & Tropical Medicine, London, UK

**Keywords:** Gametocytes, *Plasmodium falciparum*, A4, F12, *ApiAP2* gene family, Whole genome sequencing

## Abstract

**Background:**

Transmission of the malaria parasite *Plasmodium falciparum* from humans to the mosquito vector requires differentiation of a sub-population of asexual forms replicating within red blood cells into non-dividing male and female gametocytes. The nature of the molecular mechanism underlying this key differentiation event required for malaria transmission is not fully understood.

**Methods:**

Whole genome sequencing was used to examine the genomic diversity of the gametocyte non-producing 3D7-derived lines F12 and A4. These lines were used in the recent detection of the *PF3D7_1222600* locus (encoding PfAP2-G), which acts as a genetic master switch that triggers gametocyte development.

**Results:**

The evolutionary changes from the 3D7 parental strain through its derivatives F12 (culture-passage derived cloned line) and A4 (transgenic cloned line) were identified. The genetic differences including the formation of chimeric *var* genes are presented.

**Conclusion:**

A genomics resource is provided for the further study of gametocytogenesis or other phenotypes using these parasite lines.

**Electronic supplementary material:**

The online version of this article (doi:10.1186/s12936-016-1254-1) contains supplementary material, which is available to authorized users.

## Background

The malaria parasite *Plasmodium falciparum* is a major threat to human health causing approximately 200 million clinical cases per year and an estimated 438,000 deaths [1]. Malaria control efforts focus mainly on the application of insecticides to kill the mosquito vector, and on the use of anti-malarial drugs that prevent the parasite from replicating/proliferating in the liver or red blood cell stages in the host. These tools reduce the prevalence of infection, but to reach elimination additional efforts will be required to interrupt transmission. For the malaria parasite to be transmitted from humans to the mosquito vector it requires differentiation of asexually replicating forms within red blood cells into non-dividing male and female gametocytes. Only one drug available in the clinic, primaquine, can kill mature gametocytes, but this is contra-indicated in patients with G6PD deficiency. Various pharmacological agents and even anti-malarial drugs, such as pyrimethamine and chloroquine have been shown to increase gametocyte production [2–4]. Also, high asexual parasite density and diffusible factors in cultures can promote gametocyte formation [5]. Gametocytaemia is a sensitive indicator of emerging drug resistance [6]. Increasing gametocyte prevalence has been shown to precede measurable changes in parasite clearance or a decrease in cure rates. Interventions that disrupt the switch to sexual development that occurs in the human host could represent a more effective tool for malaria control and eventually elimination.

Although gametocytes were first observed microscopically in blood films taken from patients in the tropics over a 100 years ago, the molecular mechanisms involved in commitment to gametocyte formation are still poorly understood. Certain pathways have been implicated in gametocytogenesis, including those producing phorbol ester and involved in cAMP signalling [7], but these findings have not been verified using genetic approaches. Evidence has been reported that a heterotrimeric G protein based pathway may be involved [8], but the absence of homologues in the parasite genome make this unlikely. A number of studies have sought to identify loci that might constitute the trigger for the switch from asexual replication to sexual commitment. Genes in the sub-telomeric region of the right arm of chromosome 9 have been implicated in the inability to produce gametocytes, particularly the gene *PF3D7_0935400* (gametocyte development protein 1; *Pfgdv1*) [9–11]. Complementation with *PF3D7_0935400* restores gametocyte production in a 3D7 gametocyte-deficient line, which has the subtelomeric region on chromosome 9 deleted [11]. A further 16 genes associated with gametocyte production were identified by random transposon mutagenesis [12]. It has been reported that the *PF3D7_1222600* gene (a member of the *ApiAP2 f*amily encoding PfAP2-G) is an epigenetically regulated genetic master switch that triggers gametocyte development [13]. This finding was supported by several independent experimental approaches, including the identification of *Pfap2*-*g* mutations in two gametocyte non-producer (GNP) clonal lines, F12 and A4, both derived from the reference 3D7 clone. Using similar approaches the *P. berghei* AP2-G orthologue was shown to play the same role in this rodent malaria parasite [14].

The F12 strain is a well-studied GNP line [10], unable to produce either morphologically recognizable gametocytes or very early sexual stages [15]. A4 is a cloned transgenic line that was transfected with a plasmid designed to functionally delete the phosphodiesterase gene (*PDEδ, PF3D7_1470500*). A4 was generated with the intention of investigating whether the absence of *PDEδ* had any effect on exflagellation. However, the line lost the ability to produce gametocytes following drug selection, but this phenotype was unrelated to PDEδ function [16]. Both F12 and A4 clones do not have coding-sequence mutations or deletions within the sub-telomeric region of chromosome 9, nor on any other the 16 genes recently implicated in gametocyte development [13]. The only mutations found to be associated with the phenotype were those that disrupt the *Pfap2*-*g* gene [13].

Substantial genetic variation arises in clones that have been maintained in long-term culture, such as the F12 and A4 GNP lines. Genomic changes have been observed for several laboratory clones, including those derived from 3D7 [17, 18] and can be responsible for different phenotypes such as the non-production of gametocytes. Many of the mutations arising in vitro might not have an immediate selective advantage to the parasite. However in vivo, such apparently silent genetic changes might become important when a new environment (e.g., anti-malarial drugs) is presented and a selective advantage is suddenly introduced.

Here, a comprehensive analysis of the genomes of these two long-term cultured GNP lines using whole genome sequencing data and advanced computational methods is presented. The mutations identified in the two GNP lines were compared with the 3D7 Ref. [19] and with the sequence data of the parental 3D7 clone (referred here as “3D7A”) used to generate the A4 line. The data were also compared with 930 *P. falciparum* field isolates from several malaria endemic regions [20–22]. Any additional genetic changes were investigated, including structural variants and rearrangements in the *var* genes that previously have been identified in clones subjected to long-term culture. The *var* genes are the most polymorphic gene family in *P. falciparum*, with 60 loci distributed across the 14 chromosomes, both in the sub-telomeres and in internal regions. They encode the hypervariable *P. falciparum* erythrocyte membrane protein 1 (PfEMP1) that is critical for host immune evasion. Finally, a genomic resource for the two GNP lines is established, thereby enabling the research community to further study these clonal lines that have lost the ability to undergo sexual differentiation.

## Methods

### Whole genome sequencing and statistical analysis

Genomic DNA was prepared for 3D7A, F12 and A4, and underwent whole genome sequencing using Illumina technology with 76-base paired end fragment sizes [13, 23]. The raw sequence data (accession numbers ERS011445, ERS011446 and ERS011447) was processed as previously described [22]. In brief, the raw sequence data was aligned onto the 3D7 reference genome (version 3.0) using the *bwa*-*mem* short read alignment algorithm [25]. Single nucleotide polymorphisms (SNPs) and small insertions and deletions (indels) were identified using *samtools* and *GATK*, at a quality threshold of one error per one thousand basepairs (bp) [24, 25]. SNP genotypes were called using an established coverage-based approach, and polymorphisms excluded if they had missing or mixed genotypes. Larger structural variants, including deletions and amplifications were identified using alignment, sequence coverage and de novo assembly and applying the *Delly* software [26]. Unless performing candidate region analysis, we only considered genomic variants in regions that were unique (calculated by sliding 54 bp of contiguous sequence across the reference genome), non-sub-telomeric, and not in highly variable gene families (*vars, stevors, rifins*). De novo assembly was performed using *velvet* software [27], and was applied to localize the A4 plasmid insertion site. Genetic variation in the *PF3D7_1222600* gene was characterized using the tools described above for a set of 930 field isolates sequenced by the MalariaGEN *P. falciparum* community project (accession numbers ERP000190 and ERP000199) [20–22, 28]. This set included isolates from Bangladesh (n = 54), East Africa (n = 86; Kenya 17, Malawi 69), West Africa (n = 224; Burkina Faso 39, Gambia 55, Guinea-Bissau 95, Mali 35) and Southeast Asia (n = 566, Thailand 91; Cambodia 253, Lao 35; Vietnam 187). Pairwise population *F*_*ST*_ was used to assess allele frequency differences between regions, and computed using the function *stamppFst* in the R package.

### *Var* gene characterization

A coverage-based framework was used to characterize the highly polymorphic *var* gene family [15]. This approach consisted of the quantification of the total coverage and the number of *trans locus reads* across the genome using a sliding window of 100 and 25 bp overlap. *Trans locus reads* are defined as pairs of mate reads that have been improperly paired, where one of the mate pairs is located inside a *var* gene and the other mate read is located outside the same gene. A peak on the coverage in the trans locus reads together with an alteration on the total coverage (increase or decrease) in a specific region can suggest the presence of a structural variant. The structural variants found in the *var* genes using this approach were compared with those found using the *Delly* software and were annotated accordingly.

## Results and discussion

### Polymorphisms identified in gametocyte non-producer lines

**Fig. 1:**
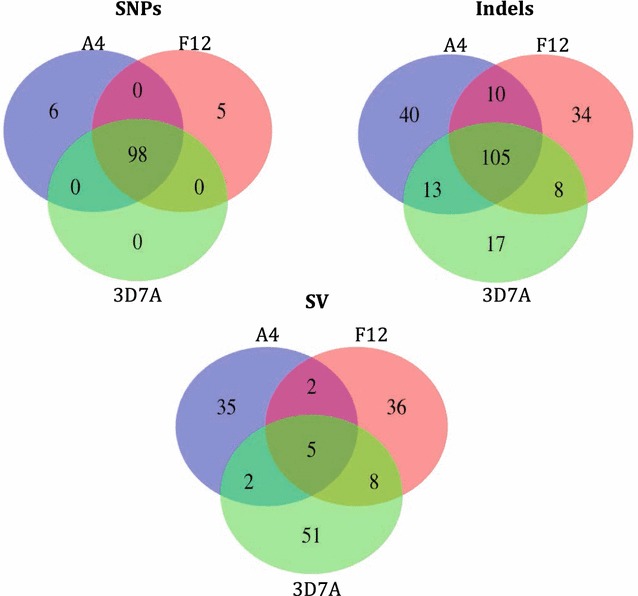
Venn diagram summarizing the polymorphisms found in A4, F12 and 3D7A clone lines. SNPs, insertions and deletions (indels) and structural variants (SVs) identified in unique and non-sub-telomeric regions of the core genome

The sequencing technology yielded in excess of thirty-six million 76 base-pair reads for each of the 3D7A, F12 and A4 cloned lines; their alignment led to an excess of 100-fold median coverage and over 98 % of the genome with at least fivefold coverage (Table 1; Fig. 1). A set of 178 high quality SNPs were identified, of which 109 (61.2 %) positions were found in unique and non-sub-telomeric regions. The majority (98/109) of SNPs had the same genotype across the 3 clones but different from the reference allele (Table 1; Fig. 1). Others have also identified differences between 3D7 clones and the reference genome [17, 18]. These discrepancies could be due to mutations introduced during long-term culture. It is also possible that some are potential sequencing or assembly errors in the 3D7 reference. Six of these SNPs cause a non-synonymous change in the protein and one introduces a stop codon in comparison to the reference (Additional file 1: Table S1). In the mitochondria and apicoplast organelles we found 2 and 12 SNPs respectively, but again the three strains had the same alleles, different from the 3D7 reference genome. No SNPs were unique for the 3D7A clone. Only 11 SNPs were unique to either two GNP strains, with no overlap in genes (Fig. 1; Additional file 1: Table S1). It was assessed whether any of these SNPs were present in a set of *P. falciparum* field isolates from countries in Africa and Asia [20–22]. Only one SNP, found in the *Pfap2*-*g* gene (*PF3D7_1222600*) that we previously described to affect gametocytes production in the F12 strain (as it introduces a stop codon) was identified in one sample from Guinea Bissau, but with a different mutation (S1308L). It is possible that this polymorphism has an effect on a gametocyte phenotype, but still allows transmission to mosquitoes. Unfortunately, no phenotypic data are available for the field isolates to study the effect of this SNP.

**Table 1:** Genome sequencing and genetic variation

Clone	Number of read pairs (Millions)^b^	Median coverage^c^	% genome with at least 5× coverage	Genetic variation^d^		
				SNPs	Indels	Structural variants
3D7A	24.6	124	99.9	98	143	66
F12^a^	24.1	118	99.9	103	157	51
A4^a^	18	132	99.9	104	168	44

^a^Gametocyte non-producers

^b^From Illumina GA II 76 bp paired-end

^c^Mapped to 3D7 version 3.0 and excluding multi-copy apicoplast and mitochondria

^d^Total variation per sample, including shared genetic variation but only polymorphisms located in non-sub-telomeric and unique regions

The polymorphism list was extended by considering short indels. A set of high quality putative small indels (140 insertions/157 deletions, median length 2, range 1–20 bp) was identified (Additional file 2: Table S2; Fig. 1). The number of indels identified in unique and non-sub-telomeric genomic regions was 118 insertions/119 deletions. Eighty-three indels were only present in A4 or F12 (47 insertions, 36 deletions). Only two genes, *PF3D7_0111200* (conserved Plasmodium protein, unknown function) and *PF3D7_0726000* (a 28 rRNA encoding gene) have indels in both lines. In the *PF3D7_0111200* gene there was an insertion in the intron. The *PF3D7_0726000* gene has one deletion and one insertion present in the two lines. The same insertion was detected in 101 field isolates of the global set and the same deletion in 112. Only ten isolates have both. The indels detected in these genes most probably do not affect the production of gametocytes, as the insertion in *PF3D7_0111200* is intronic, and the indels in the *PF3D7_0726000* gene are frequent in the field isolates (~10 %).

In this set of indels, the insertion in the *pfap2*-*g* gene in the A4 strain that results in a reading frame shift and introduction of stop codons (Additional file 2: Table S2; Chromosome 12: 913765) was identified. This is the only protein-coding gene having polymorphisms in both strains that affect the coding sequence, introducing stop codons. These polymorphisms are responsible for the non-gametocyte production phenotype, as previously described [13].

### Genetic diversity of Pfap2-g in *Plasmodium falciparum* field isolates collected across the globe

**Fig. 2:**
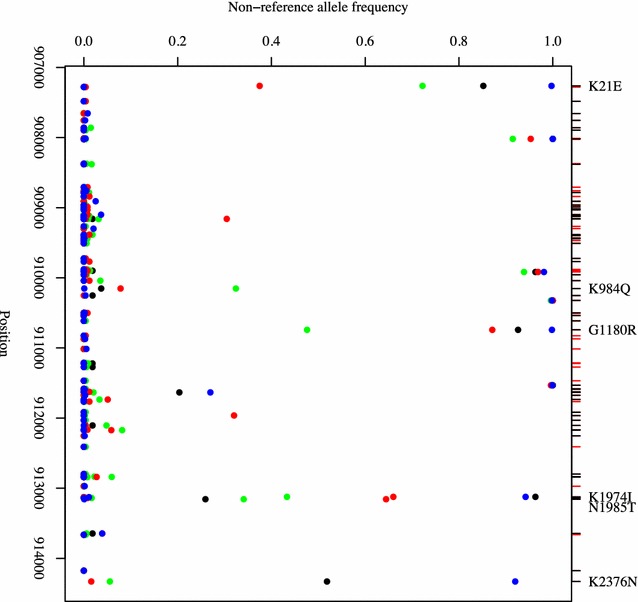
Allele frequencies of the SNPs in the *PF3D7_1222600* gene across regions: West Africa (*green*), East Africa (*red*), Bangladesh (*black*), and Southeast Asia (*blue*). All mutations shown on *upper axis* (*red tick marks* = synonymous changes, *black tick marks* = non-synonymous; *black text* = with F_ST_ values > 0.3)

A polymorphism map using a set of *P. falciparum* genomes from Africa and Asia indicated that 91 SNPs (supported by at least two isolates) reside within the *PF3D7_1222600* gene (Additional file 3: Table S3), and only 37 (41 %) are present at a minor allele frequency of more than 5 % (Fig. 2). Of these, 29 were non-synonymous mutations. Some of the mutations appear to be more common in malaria endemic regions, including the SNPs in positions 907264 (K21R, *F*_*ST*_ 0.41, East Africa vs Southeast Asia), 910153 (K984T, *F*_*ST*_ 0.35, West Africa vs Southeast Asia), 910741 (G1180E, *F*_*ST*_ 0.41, West Africa *vs* Southeast Asia), 913124 (K1974N, *F*_*ST*_ 0.31, West Africa vs Southeast Asia), 913157 (N1985 K, *F*_*ST*_ 0.53, East Africa vs Southeast Asia) and 914331 (Q2377K, *F*_*ST*_ > 0.67, Southeast Asia). Forty-six indels were identified but none change the protein frame shift nor introduce stop codons (Additional file 3: Table S3). These results emphasize the importance of this gene for parasite transmission, as no loss-of-function was detected in the field isolates.

### Structural variation in the core genome and sub-telomeric regions

**Fig. 3:**
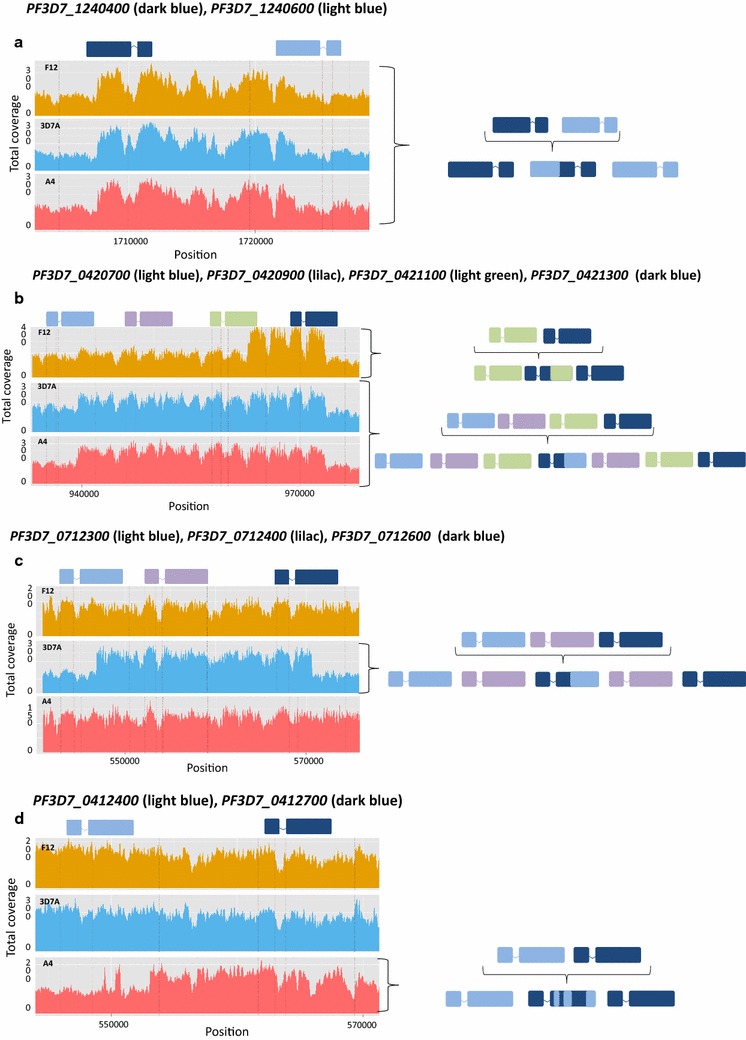
Chimeric *var* genes. **a** For all clone lines, there is twofold increase coverage for the second part of *PF3D7_1240400* (*dark blue*) and the first part of the adjacent gene, *PF3D7_1240600* (light blue). The duplication-chimera was formed by the fusion of the half of *PF3D7_1240600* and half of *PF3D7_1240400*, the *var* gene immediately adjacent. **b** For F12, a twofold increase coverage was observed for the second part of *PF3D7_0421100* (*green*), and the first part of the adjacent gene, *PF3D7_0421300* (*dark blue*) indicating a duplication-chimera formed by duplicating half of *PF3D7_0421100* and the other half of *PF3D7_0421300*. For the A4 and 3D7A strains an increased coverage of the second half of *PF3D7_0420700*, the full genes *PF3D7_0420900* and *PF3D7_0421100* and first half of gene *PF3D7_0421300,* was observed. This indicates a duplication of the genes *PF3D7_0420900* and *PF3D7_0421100* and a chimeric gene formed by duplicating half of *PF3D7_0420700* and half of *PF3D7_0421300*. **c** For 3D7A, there is twofold increase coverage for the second part of *PF3D7_0712300* (*light blue*), the whole of *PF3D7_0712400* (*lilac*) and the first part of the adjacent gene *PF3D7_0712600* (*dark blue*). It indicates a duplication of the full gene *PF3D7_0712400* and a duplication-chimera formed by duplicating half of *PF3D7_0712300* and half of *PF3D7_0712600;* **d** For A4, there is twofold increase in coverage of several regions of *PF3D7_0412400* (*light blue*) and *PF3D7_0412700* (*dark-blue*) only for A4. It indicates a chimeric gene formed by multiple cross-overs between the two adjacent genes

Deletions, translocations and amplifications of large chromosomal stretches have been observed in *P. falciparum* lines following long-term in vitro culture as well as in field isolates [17, 18]. The sequence data were examined for structural variants in these parasite lines. Few structural variants were found within the core genome (Additional file 4: Table S4; Fig. 1), consisting mostly of deletions with an average length of 250 bp, and located predominantly in non-coding regions. No truncation on chromosome 9, which has been previously implicated in the inability to produce gametocytes in vitro, was observed in these parasite lines, as previously described (Additional file 5: Figure S1) [9–11]. In the A4 strain we detected a possible deletion in a non-coding region in the telomeric region of chromosome 9 (Additional file 5: Figure S1). Rearrangements in chromosomal sub-telomeric regions were found, particularly between the *var* family gene members. Previous studies have observed recombination between sub-telomeric *var* genes involving those located either in the immediate vicinity on the same chromosome, or on different chromosomes [17, 18]. These rearrangements retain the frame-shift of the *var* genes and therefore form functional loci. Four putative rearrangements all involving neighbouring *var* genes were detected. Two chimeric genes generated by recombination between two adjacent *var* genes were identified: (i) *PF3D7_0421100* and *PF3D7_0421300* (chromosome 4) found in F12, and (ii) *PF3D7_1240400* and *PF3D7_1240600* (chromosome 12) detected in all three lines (Fig. 3a, b). Chimeric *var* genes involving exactly these pairs of adjacent genes have been reported previously in 3D7 clones [17]. More complex rearrangements were detected, including duplication of *var* genes and formation of chimeras in the same region and chimeric genes formed by multiple cross-overs in between the same two adjacent genes (Fig. 3b–d). These were only detected in single isolates. The generation of chimeric *var* genes is a critical mechanism for immune invasion, and it has been proposed that these genes have a recombination rate of up to 0.2 % per life cycle (in vitro) [17]. This means that millions of new antigenic structures could possibly be produced in a single infected individual per day, giving the parasite one-upmanship in relation to the host immune system.

Typically, var genes contain two exons. Across the three strains, we detected a highly conserved area in the intronic region between the two exons. This intronic region seems to have very high coverage because the mate sequenced paired-reads map to almost all of the other *var* genes in all chromosomes. This region has been reported previously and experimental studies have shown that the conserved *var* introns are important regulatory elements [29, 30]. The regulation of *var* gene expression and silencing is closely linked to the regulation of sexual commitment, and both mechanisms are controlled by the same epigenetic machinery. The knockdown of the heterochromatin protein 1 (*PF3D7_1220900*) and a histone deacetylase (*PF3D7_1008000*) promote gametocytogenesis and leads to de-regulation of mutually exclusive *var* gene expression [30–32]. It is not known whether disruptions in *va*r gene expression, due to e.g., chromatin modifications, also affect *var* gene recombination and, therefore, antigenic diversity. It has been suggested that introducing malaria control programs may increase parasite virulence in some circumstances [33]. The share of the core epigenetic machinery between these fundamental parasitic processes should be taken into account during control interventions (e.g., vaccine introduction) as blocking one process might unexpectedly affect the other process.

### Location of the A4 plasmid insertion sites following transfection and drug selection

The A4 is a cloned transgenic line that was originally generated with the intention of investigating whether the absence of the phosphodiesterase gene (*PDEδ*, *PF3D7_1470500*) had any effect on exflagellation. The A4 clone harbours a whole plasmid (7 kbp; more than one copy) designed to delete the *PDEδ* gene [34]. Following drug selection, this transgenic line lost the ability to produce gametocytes, but this phenotype was unrelated to PDEδ function. Importantly, it was subsequently shown using another cloned line in which the *PDEδ* gene had been successfully disrupted that whilst the production of gametocytes was unaffected, there was a severe reduction in the ability to undergo gametogenesis [16]. The A4 sequence data was used to confirm the genomic insertion of the plasmid and the possibility that integration happened at more than one site and disrupted other genes. De novo assembly of the reads led to a contig of length ~10 kbp, containing the ~7 kbp intact plasmid (derived from pHTK [35]), and a flanking 3 kbp region. A blast search of the contig to the reference genome indicated that the *P. falciparum*-derived elements of the plasmid matched to their respective chromosome sites with at least 99 % identity. In particular, the 3D7 derived contig regions 1–845, 5510–6062, 6269–7134, 7360–10303 matched to chromosome 14 (2883802-2884646, *PDEδ*), 7 (381591-381037, *HSP90*), 14 (2882276-2883143, PDEδ), and 14 (1369973–1372928, *PF3D7_1434300*, HOP—Hsp70/Hsp90 organizing protein), with 99.8, 98.7, 99.1 and 99.5 % identity, respectively. Pulse field blot analysis of A4 indicated an insertion of the plasmid within chromosome 14 [34], leaving the *PDEδ* (located in 2882766–2886082) and *HOP* (*PF3D7_1434300* located in 1372903–13745970) regions as potential candidates for insertion. Regions neighbouring the HOP and PDEδ (2882276–2883143) genes showed an excess of read coverage suggesting potential insertion sites for the plasmid (Additional file 6: Figure S2). To localize the exact plasmid integration site, the 3 kbp of the plasmid flanking sequence generated when performing de novo assembly of the plasmid was used. This flanking region contains pair-reads that mapped both the plasmid and the *HOP* region (1369961–1372928), confirming this location as a likely site of plasmid integration (Additional file 6: Figure S2). Excess coverage detected in two PDEδ regions corresponds to the two sequences that were inserted into the vector.

This sequence data analysis indicates that it is possible that integration occurred in a different genomic location to that intended, and that care must be taken when interpreting the observed phenotype. The application of whole genome sequencing and new reverse genetic methodology will ensure such issues will be less problematic in future.

### Prediction of gametocyte/sexual development genes using DNA binding sequence motifs

A large proportion of *P. falciparum* genes lack a known function. Using a bioinformatic approach it is possible to identify genes that could function at specific *P. falciparum* developmental stages, and take into account similarities in gene expression patterns to predict function (‘guilt by association’). By applying this method, a study using stage-specific gametocyte mRNA preparations in conjunction with microarray analysis and using ‘guilt by association’ approach, generated a ‘sexual development’ cluster containing 246 genes [36]. A palindromic sequence (TGTANNTACA) was identified in the 5′ UTR of 65 genes in this cluster [36]. The A4 and F12 whole genome sequence data were scanned for this motif and 23 genes were found, seven overlapping with the 65 in the cluster. There was also a scan for the consensus DNA-binding preference of the transcription factor PfAP2-G that binds to specific motifs in the 5′ upstream region of early gametocyte genes to turn on transcription [37]. Fifty-one genes were identified, 11 also having the palindromic sequence and five of them overlapping with the 65 genes in the sexual development cluster (Additional file 7: Table S5). According to Illumina-based sequencing of *P. falciparum* 3D7 mRNA from gametocytes and erythrocytic stages [38], these 11 genes have higher expression during the sexual stages. Three of these genes were present in the guilt by association data set. This overlap could suggest that the two motifs, identified by independent means, may have a relationship in terms of determining timely expression of genes, particularly at the onset of gametocyte/sexual development, as the transcription factor PfAP2-G seems to regulate the expression of early gametocyte genes.

The small overlap between the various datasets may indicate that other regulatory motifs besides the two analysed here, are important during the sexual stage. Overall, these types of approach, that integrate data from varying bioinformatics and gene expression studies, are important to identify target genes for further functional work, particularly when so many genes in the *P. falciparum* genome are hypothetical proteins. However, several genes may be missed, especially if they have low expressions levels, are regulated by different mechanisms or involved in negative regulation. Moreover, there is the need to support gene transcription data particularly with quantitative proteomics, to generate more robust hypotheses.

## Conclusion

A comprehensive analysis is presented of the three *P. falciparum* genomes that were previously used as part of a study to determine that the transcriptional regulator AP2-G is a key determinant of sexual differentiation. These cloned lines have been in long-term culture, so as expected several SNPs, indels, structural variants and some new chimeric *var* genes were identified. The acquisition of genetic variants and the recombination of the *var* genes during mitosis provide the parasite with a mechanism that can generate an enormous amount of genetic diversity, which could lead to selective advantage. These mutations might be phenotypically undetectable or may cause a visible phenotype, as in the case of the gametocyte non-production in the F12 and A4 clones. Also, by performing whole genome sequencing of a transgenic line we have identified an unexpected integration site. This finding may motivate others to take this step to identify any additional changes that have occurred in transgenic lines that might potentially contribute to a phenotype. These lines were fundamental to identifying the *Pfap2*-*g* gene, and they provide an important tool to understand gametocytogenesis in vitro. There are still outstanding questions concerning how sexual commitment occurs in vivo, and whether this locus may be useful in predicting and monitoring transmission. Ultimately, fully understanding the molecular events underpinning gametocyte production could lead to a therapeutic which totally prevents sexual development and thereby blocks transmission. The A4 and F12 gametocyte non-producing lines and their sequence data will be a useful resource for future studies looking to translate biological insights into malaria control measures.

## Availability of supporting data

The sequence data were generated by the Malaria Programme at the Wellcome Trust Sanger Institute and are publically available from the EBI short read archive (accession numbers: Clonal strains ERS011445, ERS011446 and ERS011447; field isolates ERP000190 and ERP000199). The data can also be accessed and browsed through the Pf3k web application (www.malariagen.net/apps//pf3k).

## Additional files

10.1186/s12936-016-1254-1 SNPs identified in the three lines 3D7A, A4 and F12.
10.1186/s12936-016-1254-1 Insertions (INS) and deletions (DEL) identified in the three lines 3D7A, A4 and F12.
10.1186/s12936-016-1254-1 SNPs and Indels identified in the *PF3D7_1222600* gene among *P. falciparum* field isolates from Africa and Asia.
10.1186/s12936-016-1254-1 Structural variants identified in the three lines 3D7A, A4 and F12.
10.1186/s12936-016-1254-1 Coverage plots to evaluate evidence of truncation in the end of chromosome 9. (a) Coverage in the chromosome 9 candidate region (20 genes from *PF3D7_0935400* to *PF3D7_0937300,* shaded) showing no evidence of deletion (also confirmed using CGH data (not presented)) (A4, blue; 3D7A, green; F12, red); (b) Evidence of a deletion in a sub-telomeric non-coding region of chromosome 9 in the A4 strain, but not 3D7A or F12.
10.1186/s12936-016-1254-1 Coverage plots showing potential insertion points of the A4 plasmid. Regions neighbouring the (a) *HOP* and (b) *PDE*-*delta* genes showed an excess of read coverage suggesting potential insertion sites for the plasmid. The flanking region of the *de novo* assembled plasmid-contig maps to the *HOP* region confirmed this location as a site of plasmid integration (Chromosome 14: 1369961–1372928). Excess coverage observed in two PDEδ regions corresponds to the two sequences that were inserted into the vector.
10.1186/s12936-016-1254-1 A scan for motifs in gene sequences (coding and UTR regions).

## Abbreviations

SNPs: single nucleotide polymorphisms
GNP: gametocyte non-producer
indels: insertions and deletions
bp: basepairs
SVs: structural variants
