# Supplementary figures and images for: Genomic variation in two gametocyte non-producing Plasmodium falciparum clonal lines
Source: Malar J. 2016 Apr 21;15:229. doi: 10.1186/s12936-016-1254-1 (PMC4839107; doi:10.1186/s12936-016-1254-1)

**(a)**

**
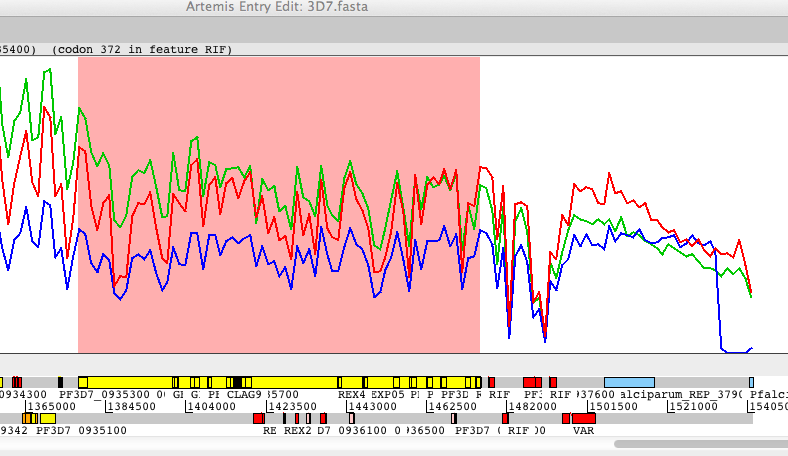
**

**(b)**

**
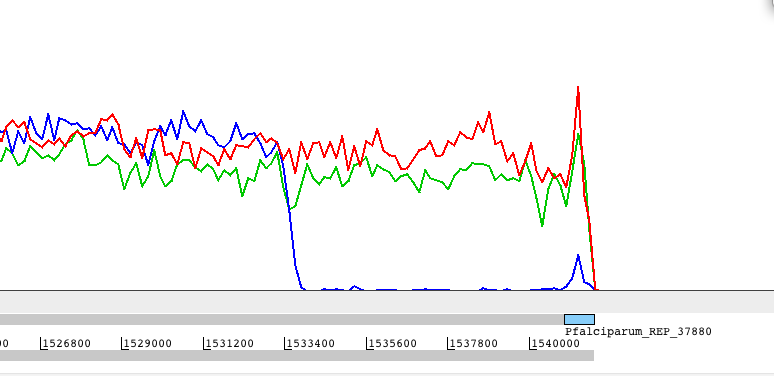
**

Supplement: Supplementary file 5 — 10.1186/s12936-016-1254-1 Coverage plots to evaluate evidence of truncation in the end of chromosome 9. (a) Coverage in the chromosome 9 candidate region (20 genes from PF3D7_0935400 to PF3D7_0937300, shaded) showing no evidence of deletion (also confirmed using CGH data (not presented)) (A4, blue; 3D7A, green; F12, red); (b) Evidence of a deletion in a sub-telomeric non-coding region of chromosome 9 in the A4 strain, but not 3D7A or F12. [file 12936_2016_1254_MOESM5_ESM.docx]

**(a)**


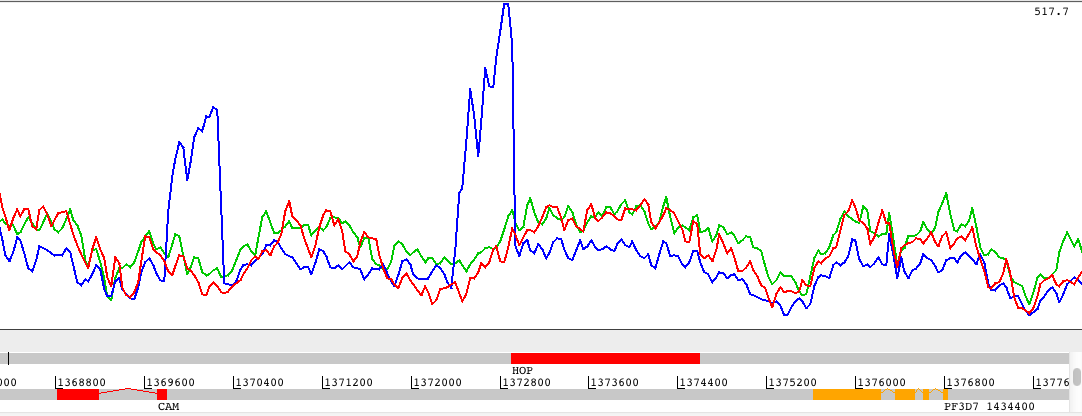


**(b)**

**
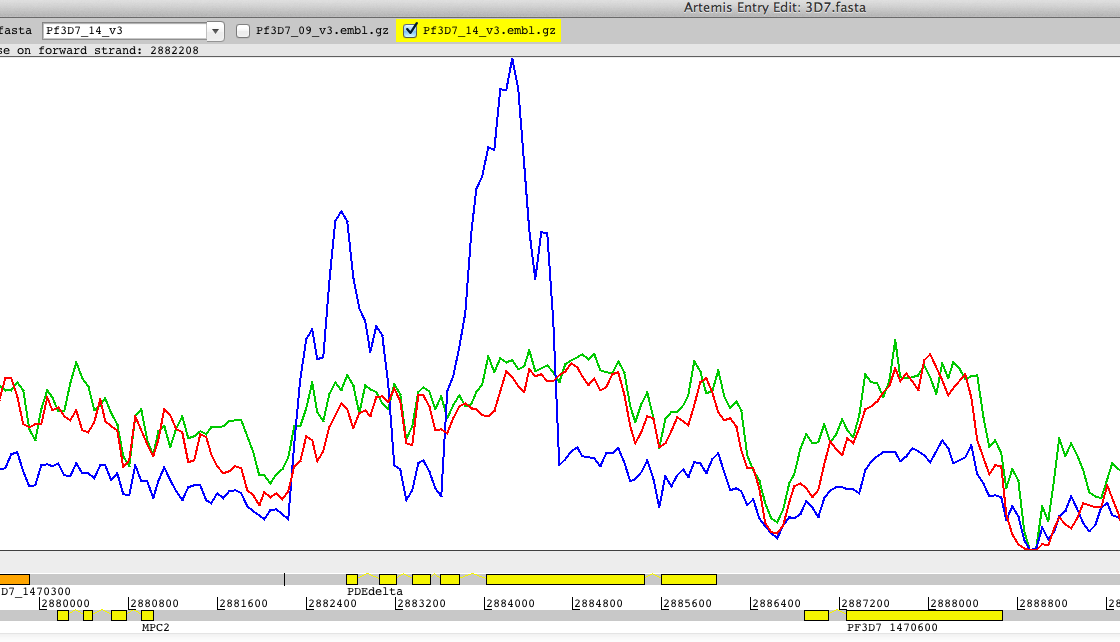
**

Supplement: Supplementary file 6 — 10.1186/s12936-016-1254-1 Coverage plots showing potential insertion points of the A4 plasmid. Regions neighbouring the (a) HOP and (b) PDE-delta genes showed an excess of read coverage suggesting potential insertion sites for the plasmid. The flanking region of the de novo assembled plasmid-contig maps to the HOP region confirmed this location as a site of plasmid integration (Chromosome 14: 1369961–1372928). Excess coverage observed in two PDEδ regions corresponds to the two sequences that were inserted into the vector. [file 12936_2016_1254_MOESM6_ESM.doc]
